# An Unexpected Twist: Sigmoid Volvulus Complicated by Peritoneal Metastases

**DOI:** 10.7759/cureus.82570

**Published:** 2025-04-19

**Authors:** Aabid Mohiuddin, Fawaz Hussain, Eric Denha, Christine Schad, Fadi Antaki, Samara Rifkin

**Affiliations:** 1 Department of Internal Medicine, Detroit Medical Center/Wayne State University, Detroit, USA; 2 Department of Gastroenterology, Detroit Medical Center/Wayne State University, Detroit, USA; 3 Department of Surgery, Henry Ford Hospital, Detroit, USA; 4 Department of Gastroenterology, John D. Dingell Veterans Affairs Medical Center, Detroit, USA

**Keywords:** endoscopic detorsion, pancreatic adenocarcinoma, peritoneal metastases, recurrent volvulus, sigmoid volvulus

## Abstract

Sigmoid volvulus is the mechanical torsion of the sigmoid colon, its mesentery, and blood supply around itself, resulting in luminal obstruction and colonic ischemia. Initial management typically involves endoscopic detorsion; however, patients with peritonitis or who fail endoscopic therapy warrant surgical intervention. This case illustrates the rare presentation of recurrent sigmoid volvulus, which was refractory to endoscopic detorsion due to complications of peritoneal metastases, which tethered the sigmoid into a torsed position, ultimately requiring surgical resection. This report emphasizes the need for tailored, collaborative approaches to managing sigmoid volvulus in patients with underlying malignancy.

## Introduction

Sigmoid volvulus is an uncommon yet potentially fatal cause of intestinal obstruction, accounting for less than 10% of large bowel obstruction noted among adults in the United States [1,2]. ⁤⁤It is characterized by torsion of the sigmoid colon around its own supporting mesentery, causing significant abdominal pain, distress, distention, and constipation due to luminal obstruction and impairment of intestinal perfusion. ⁤⁤This can subsequently result in bowel ischemia, gangrenous necrosis, and perforation without prompt endoscopic or surgical intervention [3].

The initial treatment of choice for uncomplicated acute sigmoid volvulus is endoscopic detorsion. However, the presence of high-risk features - such as diffuse peritonitis or perforation - typically warrants emergent surgical intervention [4,5]. In non-emergent cases managed endoscopically, elective sigmoid colectomy with primary colorectal anastomosis is recommended during the same admission for definitive management [6]. The following case illustrates the rare clinical presentation of sigmoid volvulus refractory to multiple endoscopic detorsion attempts due to peritoneal metastatic lesions pinning the sigmoid colon into a torsed position and the importance of surgical collaboration.

## Case presentation

**Figure 1:**
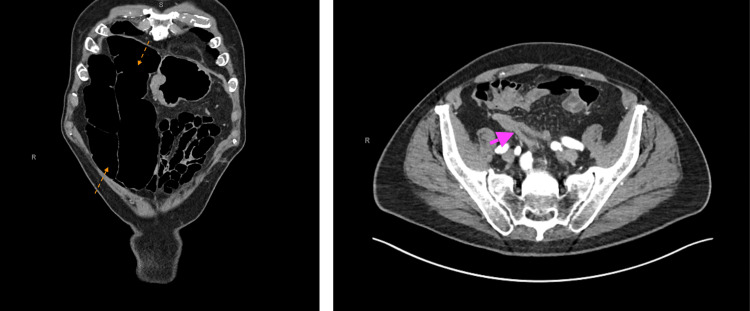
On the coronal view (left), CT imaging of the abdomen revealed a large volvulus, while the axial view (right) reveals the transition point at the sigmoid volvulus. Orange arrows depict the dilated bowel loops caused by the volvulus. The pink arrow depicts the transition point at the sigmoid volvulus.

An 84-year-old man with a prior history of stage 1b pancreatic ductal adenocarcinoma presented with five days of abdominal distention, pain, and constipation that occurred two months after distal pancreatectomy for a tail pancreatic mass at an outside institution. He was not acutely distressed; on examination, his abdomen was soft, mildly tender to palpation diffusely, and without guarding or rebound tenderness. His initial laboratory values in the emergency department revealed hyperlactatemia (4.2 mmol/L); however, it improved to normal range after fluid resuscitation. There was no leukocytosis, and other relevant markers (liver enzymes, hemoglobin, etc.) were within normal range (Table 1). Computed tomography (CT) imaging of his abdomen with contrast revealed sigmoid volvulus (Figure 1). Following evaluation by gastroenterology and general surgery, endoscopic detorsion was recommended. Flexible sigmoidoscopy achieved complete detorsion, which led to marked symptomatic improvement. Notably, the patient declined the recommended elective sigmoidectomy.

**Table 1:** Relevant lab values from the patient's two admissions for abdominal pain. ALT: alanine aminotransferase; AST: aspartate aminotransferase; ALP: alkaline phosphatase

Laboratory parameters	Initial admission	Subsequent admission (4 months later)
ALP (reference range: 40-129 units/L)	84	124
ALT (reference range: 7-55 U/L)	30	42
AST (reference range: 8-48 U/L)	28	22
Total bilirubin (reference range: 0.1-1.2 mg/dL)	0.4	0.8
Lipase (reference range: 0-160 U/L)	24	28
Lactic acid (reference range: 0.7-2.1 mmol/L)	4.2	1.6
White blood cell count (reference range: 3.5-10.6 units x 1000/uL)	4.2	5.3
Hemoglobin (reference range: 13.3-17.1 g/dL)	13.3	14.7

**Figure 2:**
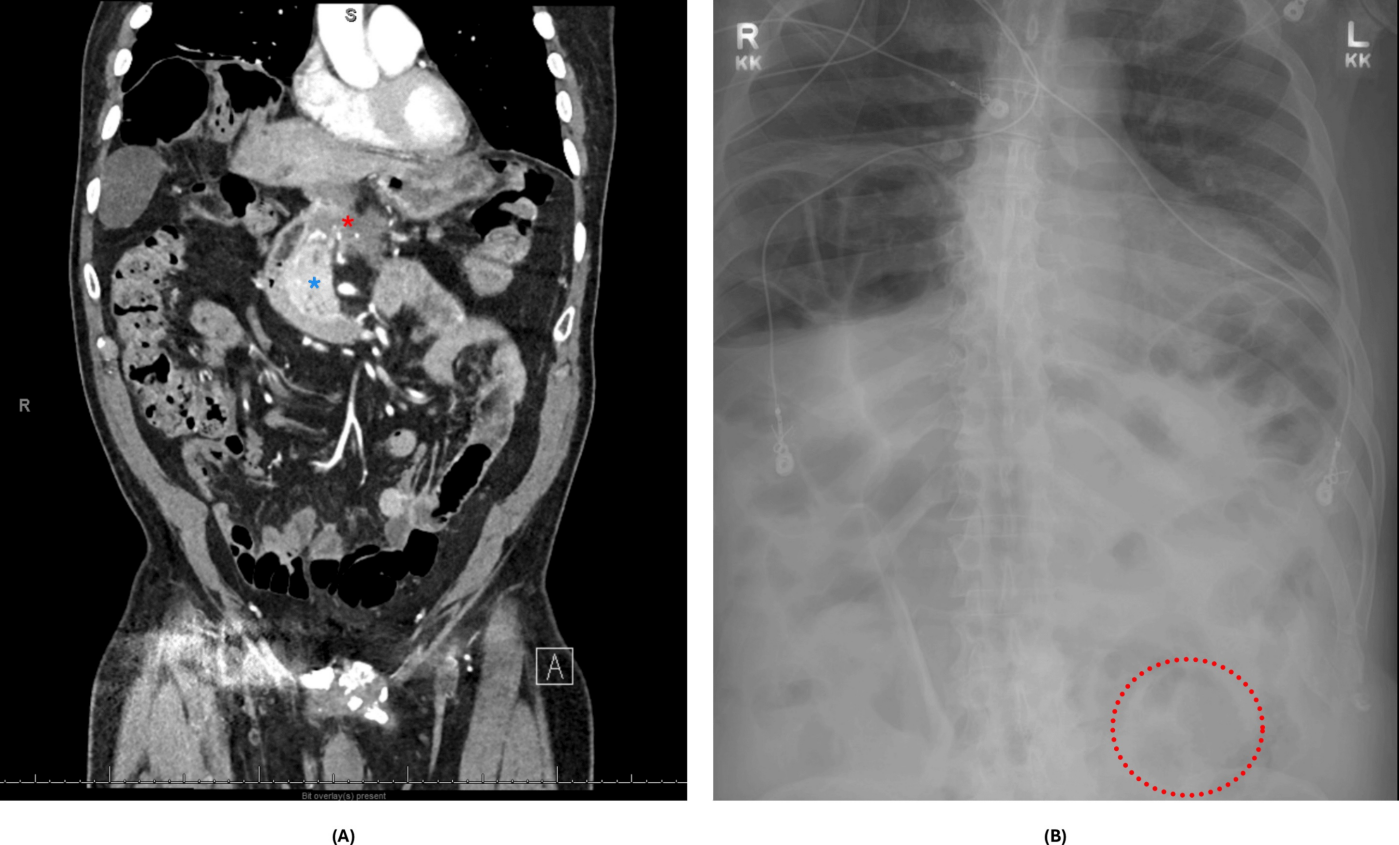
(A) CT imaging revealed increased soft tissue around the distal pancreatectomy site, concerning for malignancy. (B) An abdominal radiograph obtained post-endoscopic detorsion showed ongoing, re-demonstrated volvulus. (A) The blue star denotes the pancreas. The red star denotes a relatively hypo-attentuated area suggestive of soft tissue mass. The small hyper-attenuated spots in the mass are surgical clips from the previous distal pancreatectomy. (B) The red circle highlights the "coffee-bean sign," which is a radiologic sign of sigmoid volvulus.

Four months later, he returned with identical symptoms and similar physical exam and laboratory findings (Table 1). CT imaging revealed recurrent sigmoid volvulus as well as interval increased soft tissue around the distal pancreatectomy site concerning for recurrent pancreatic adenocarcinoma (Figure 2A). The patient underwent flexible sigmoidoscopy with successful endoscopic decompression. However, an abdominal X-ray obtained following the procedure showed ongoing, re-demonstrated volvulus (Figure 2B).

**Figure 3:**
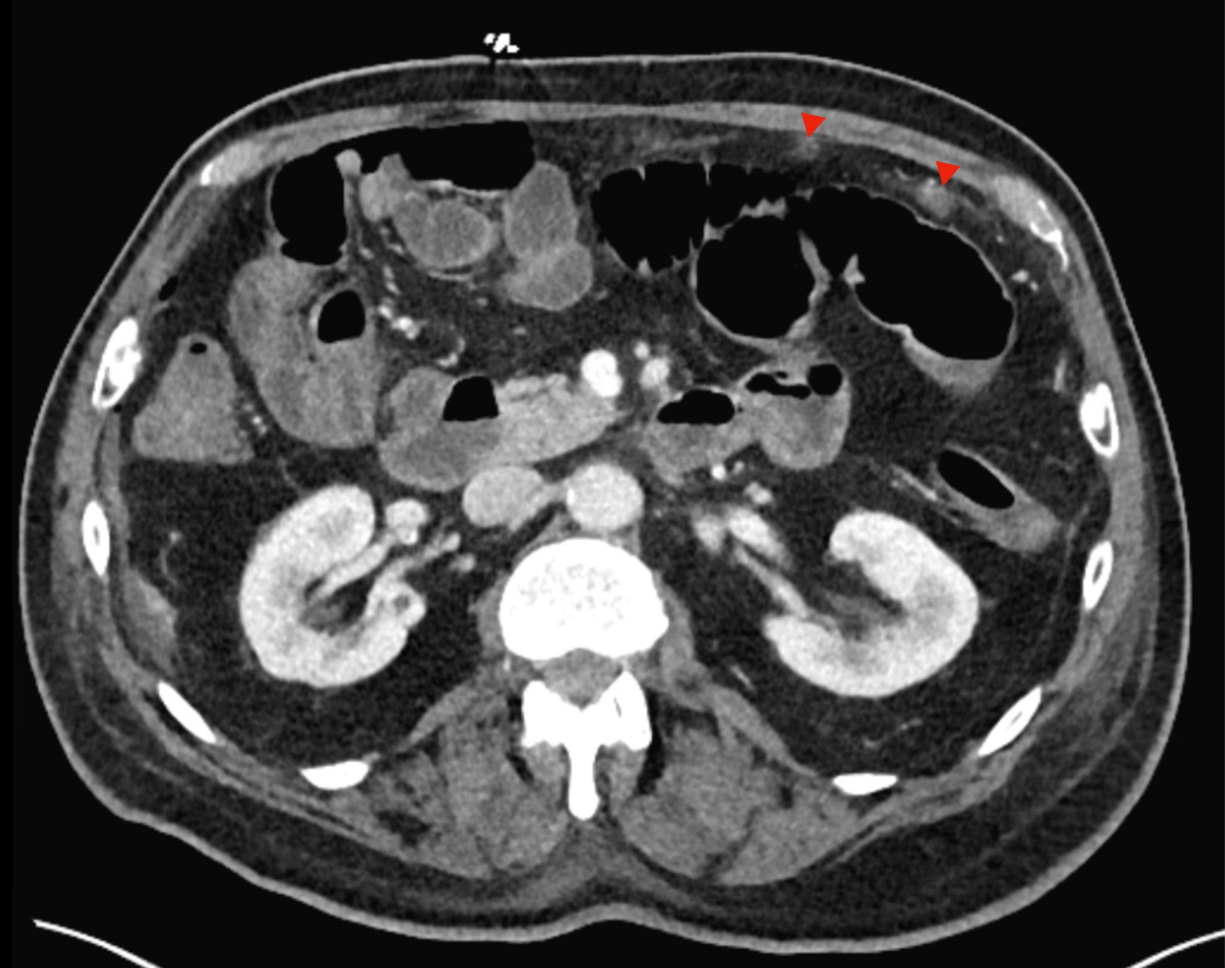
Post-operative computed tomography (CT) imaging (obtained with intravenous contrast) revealed multiple omental nodules which further reinforced the operative findings of diffuse peritoneal carcinomatosis. The red arrows highlight two separate omental nodules.

Due to the inability to detorse the volvulus endoscopically, the patient was advised to undergo surgical sigmoid resection. He initially refused surgery; however, he ultimately agreed to proceed if an additional attempt to detorse the volvulus was unsuccessful. Our GI team had closely collaborated with surgery given the patient’s increasing need for surgery and his reticence to complete it. Therefore, this flexible sigmoidoscopy was performed in the operating room with the surgical team present. After the attempt to endoscopically detorse the volvulus proved unsuccessful, the surgery team was able to immediately and seamlessly transition to a surgical approach. Notably, the colon did not eviscerate upon abdominal entry and remained tethered with attempted manual evisceration. The proximal sigmoid colon traversed the midline with its mesentery pinned to the terminal ileal mesentery by a hard, nodular point of fixation. Numerous diffuse nodular deposits were palpated throughout the abdomen. Ultimately, sigmoid colectomy with end colostomy was performed. After a few days, abdominal CT imaging was obtained to evaluate the colostomy site post-operatively and revealed multiple omental nodules concerning for peritoneal carcinomatosis (Figure 3). The deposits were later confirmed on biopsy as metastatic lesions from the primary pancreatic adenocarcinoma. After his post-operative course, the patient was discharged home, ultimately electing for enrollment in home hospice.

## Discussion

Sigmoid volvulus most often occurs in older men, with a mean age of 70 years old [2]. Typically, it presents in the setting of an elongated, redundant sigmoid colon - either due to existing colonic dysmotility (i.e., chronic constipation) or an anatomical predisposition to increased length of the sigmoid colon [7-9]. In addition, small-bowel obstruction or abdominal adhesions from prior abdominal surgeries can act as points of fixation in causing sigmoid volvulus [4].

In this unique case presentation, the sigmoid colon was tethered into a torsed position by a peritoneal metastatic nodular lesion. Upon review of the current literature, there is only one other similar reported case that details volvulus complicated by peritoneal carcinomatosis; however, this case details small bowel volvulus due to metastatic gastric signet ring cell adenocarcinoma [10]. Thus, to our best knowledge, this case is the first to report recurrent sigmoid volvulus provoked by peritoneal metastasis from pancreatic ductal adenocarcinoma.

In typical management of uncomplicated sigmoid volvulus, elective sigmoidectomy is strongly recommended after successful endoscopic detorsion is achieved, as it has proven superior to non-resectional procedures (i.e., sigmoidopexy or mesosigmoidopexy) [11]. For patients who did not receive elective sigmoidectomy after initial endoscopic detorsion, the volvulus recurrence rate is 84%, with a median time to recurrence a mere 58 days [12].

This case highlights the need for further considerations when managing sigmoid volvulus in patients with known or suspected malignancy. Repeated inability to achieve endoscopic detorsion of the volvulus despite multiple attempts should prompt consideration of mechanical constraint caused by peritoneal metastases, as seen in this case. Early recognition and intervention can help minimize the risk of progression to bowel ischemia or perforation. As with the management of uncomplicated sigmoid volvulus, emphasizing the importance of elective sigmoidectomy in patients with malignancy is crucial to preventing recurrence.

## Conclusions

Overall, this case contributes to our understanding of this rare complication of sigmoid volvulus seen in patients with metastatic cancer. It also underscores the importance of close multidisciplinary collaboration between endoscopists and surgeons to facilitate a comprehensive management approach. The current scarcity of information on the management of recurrent volvulus in patients with malignancy calls for further studies to help guide clinicians in tailoring interventions to improve outcomes in this patient population.
